# Bat organoid platform: revolutionizing zoonotic virus research and pandemic preparedness

**DOI:** 10.1038/s41392-025-02323-7

**Published:** 2025-07-16

**Authors:** Young-Ho Park, Sun-Uk Kim, Taeho Kwon

**Affiliations:** 1 Futuristic Animal Resource and Research Center, Korea Research Institute of Bioscience and Biotechnology (KRIBB), Cheongju, Chungbuk Republic of Korea; 2 Advanced Bioconvergence Department, Department of Functional Genomics, KRIBB School, Korea National University of Science and Technology (UST), Daejeon, Republic of Korea; 3 Primate Resources Center, Korea Research Institute of Bioscience and Biotechnology (KRIBB), Jeongeup, Jeonbuk Republic of Korea

**Keywords:** Infectious diseases, Molecular medicine

A recent study published in *Science* by Hyunjoon Kim and colleagues has established the world’s most comprehensive bat organoid platform, marking a significant advancement in comparative virology and pandemic preparedness research.^1^ This innovative research addresses critical knowledge gaps in zoonotic disease transmission by developing sophisticated laboratory models that closely mimic bat tissues.

**Fig. 1:**
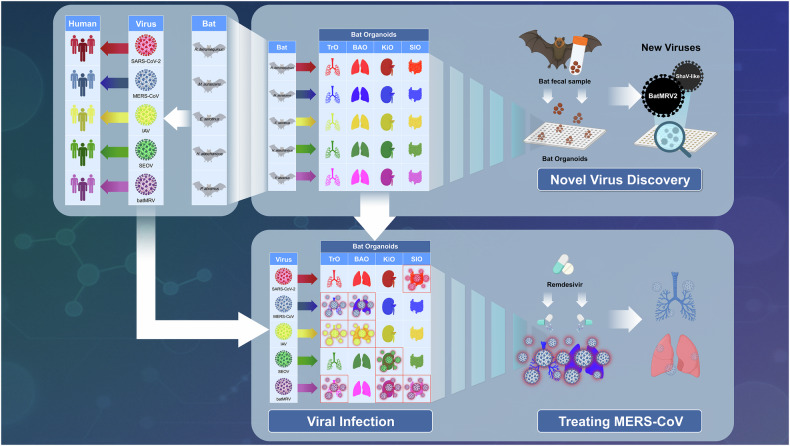
Bat organoid platform for virus research and discovery. The schematic illustrates the workflow and utility of the multispecies bat organoid platform for studying virus-host interactions, antiviral testing, and discovery of previously unknown pathogens. Initially, epithelial organoids representing various tissues bronchoalveolar (BAO), trachea (TrO), kidney (KiO), and small intestine (SIO) are generated from multiple bat species. These organoids closely mimic the cellular architecture and complexity of the corresponding bat tissues. The upper panel depicts how field-collected bat fecal samples can be introduced into bat organoids to isolate and propagate novel viruses, such as mammalian orthoreovirus (MRV) and Shaanvirus-like paramyxovirus. The lower panel illustrates how the organoid platform facilitates comparative infection studies, revealing species-specific and tissue-specific tropisms for zoonotic pathogens, including SARS-CoV-2, MERS-CoV, influenza A viruses (IAV), and Orthohantavirus Seoulense (SEOV). Furthermore, the platform supports high-throughput antiviral screening; for example, remdesivir demonstrated greater antiviral efficacy in organoid models compared to traditional cell lines, underscoring the translational relevance and specificity of bat-derived organoids for therapeutic development. This integrative approach significantly enhances our understanding of viral pathogenesis, cross-species transmission risks, and pandemic preparedness. The figure was created with BioRender.com

The research team successfully generated organoids representing four essential organ systems—airways, lungs, kidneys, and small intestine using cells from five phylogenetically diverse bat species across Asia and Europe.^1^ The five examined bat species (*Rhinolophus ferrumequinum*, *Myotis aurascens*, *Pipistrellus abramus*, *Eptesicus serotinus*, and *Hypsugo alaschanicus*) are East Asian insectivorous bats with broad Eurasian distributions that harbor zoonotic pathogens, including coronaviruses, paramyxoviruses, and reoviruses. This multi-organ organoid platform provides a methodological framework applicable to other key bat species (e.g., Pteropus spp.) and geographically diverse populations, supporting global virome surveillance and pandemic preparedness. This comprehensive organoid platform facilitates systematic investigation of virus-host tissue interactions under a controlled laboratory environment (Fig. 1).

Given that zoonotic viruses such as Nipah and Ebola transmit from bats to humans through direct contact or contaminated sources, incorporating Pteropus organoids the natural reservoir of Nipah virus, would significantly enhance our understanding of bat-to-human transmission mechanisms. The inclusion of respiratory, digestive, and urinary tract organoids represents a key methodological advantage, enabling representative modeling of viruses with distinct tissue tropisms. This approach allows pathogen-specific organoid selection for targeted investigations, such as respiratory models for influenza or renal organoids for hantaviruses, thereby enhancing experimental relevance and translational potential.

Using cutting-edge techniques, including multiplexed immunohistochemistry, single-cell RNA sequencing, and transmission electron microscopy, the researchers meticulously characterized their organoid models. The original study established a comprehensive transcriptomic baseline for organoids using singlecell RNA sequencing on *R. ferrumequinum* and *E. serotinus* models, confirming cellular diversity and faithful recapitulation of native tissue architecture. However, the absence of high-quality reference genomes for the four Vespertilionidae species represents a significant limitation, highlighting the critical need for enhanced genomic resources to fully exploit these models for comparative host-pathogen interaction studies. These analyses revealed that the lab-grown tissues closely replicated the complexity of actual bat organs. One particularly fascinating discovery was the near absence of MUC5AC-positive goblet cells across all bat species studied. This finding suggests an evolutionary adaptation where bats have minimized mucus production in favor of enhanced airborne immune defenses, a strategy that may contribute to their unique ability to harbor viruses without becoming severely ill.

The researchers systematically tested how major zoonotic pathogens, including SARS-CoV-2, MERS-CoV, influenza A viruses, and hantaviru,s infected different bat organs. Crucially, these infection patterns correlated with tissue-specific expression of key viral entry receptors, including ACE2 for SARS-CoV-2, DPP4 for MERS-CoV, and α2,3- and α2,6-linked sialic acid receptors for influenza A virus, thereby determining viral tropism across the organoid panel. Distinct entry receptors and host determinants shape virus-specific tropism: SARS-CoV-2 and influenza A viruses preferentially infect intestinal and respiratory tissues, respectively, while hantavirus targets renal organoids. These tropisms recapitulate key pathological lesions observed in humans and other mammals. Organoid models reveal species- and tissue-specific differences, elucidating fundamental mechanisms of cross-species transmission and viral pathogenesis. The results revealed complex, species-specific infection patterns that challenge our understanding of viral tropism. Despite the presence of ACE2 receptors (the primary entry point for SARS-CoV-2), the virus failed to infect respiratory organoids but successfully infected intestinal tissues from *R. ferrumequinum*. This finding aligns with previous observations and suggests that receptor presence alone doesn’t guarantee infection. MERS-CoV demonstrated wider tissue tropism, successfully infecting lung organoids from multiple bat species. The infection efficiency directly correlated with the expression levels of DPP4 receptors, providing valuable insights into viral entry mechanisms.^1,2^ Influenza A viruses showed remarkable respiratory tropism across bat species due to the co-expression of both avian (α2,3-linked) and human (α2,6-linked) sialic acid receptors. Notably, bat-derived H9N2 strains infected tissues as efficiently as human and avian isolates, highlighting the potential for cross-species transmission. A crucial finding was that the organoid platform maintained functional innate immune responses. RNA sequencing following viral infections revealed robust activation of interferon-stimulated genes (ISGs), with distinct response patterns varying by both virus type and bat species.^1,3^ These results suggest that MERS-CoV triggered weaker immune responses compared to influenza A virus, suggesting different viral immune evasion strategies.^1^

To assess translational utility, 3D organoids were adapted to 2D monolayers in 96-well plates for antiviral high-throughput screening. Comparative drug testing revealed that Remdesivir significantly inhibited viral replication in bat organoids while showing limited efficacy in conventional Vero E6 cell lines, highlighting the platform’s potential for identifying host-specific therapeutic targets and pharmacodynamic profiles.^1,3,4^ In addition to the screening for known pathogens, the platform has also successfully isolated and characterized previously unknown bat viruses from fecal samples, including mammalian orthoreovirus (MRV) and Shaanvirus-like paramyxovirus. These newly discovered viruses showed productive replication and distinct cellular damage patterns, with genetic analysis revealing complex viral evolution patterns. The bat organoid platform presents a novel system to investigate retroviruses, particularly endogenous retroviral elements (ERVs). Following their recent identification in iPS cells,^5^ determining the expression and activity of ERVs in bat organoids could illuminate their evolutionary dynamics, functional consequences, and potential interplay with exogenous viruses. In addition, the organoid platform proved remarkably effective at modeling tissue-specific viral interactions. Cross-species studies showed that *M. aurascens* organoids from multiple organs supported both H1N1 and H5N1 infections, effectively replicating the widespread viral distribution seen in severe influenza cases. Similarly, kidney organoids from certain bat species (*R. ferrumequinum* and *E. serotinus*) exhibited susceptibility to Orthohantavirus Seoulense (SEOV), mirroring the kidney damage observed in human hantavirus infections. The virus triggered cell death and tissue barrier breakdown in the lab models, confirming their relevance for understanding disease mechanisms.^1,4^

Recent advances have provided high-quality draft genomes and transcriptomic datasets for the five bat species used in the study. These genomic and transcriptomic resources are essential for interpreting virus-host interactions at the molecular level, enabling comparative analyses, and facilitating targeted antiviral drug screening in bat organoid models.

Despite these significant advances, several constraints limit the platform’s full potential. Genomic annotation remains challenging due to incomplete reference genomes for many bat species, complicating precise functional analyses. To address this limitation, future genomic sequencing initiatives combined with advanced bioinformatics tools, potentially enhanced by emerging computational approaches such as quantum computing or quantum biological convergence, could significantly improve genomic annotation accuracy and deepen functional characterization of bat-specific genes. However, a critical limitation is the lack of immune cell components, precluding a comprehensive assessment of innate-adaptive immune interactions essential to understanding bat-virus evolutionary dynamics. Immunological modeling represents another critical gap, as current organoid systems lack immune cell populations necessary to model epithelial-immune interactions accurately. Incorporating dendritic cells, macrophages, and lymphocytes into the organoid cultures would substantially enhance physiological relevance by enabling detailed investigations of antigen presentation, cytokine signaling, and adaptive immune responses critical for viral clearance and immunopathology. Additionally, the current exclusive focus on epithelial tissues overlooks other essential tissue compartments. Expanding the platform to include endothelial, neuronal, and stromal organoids would greatly broaden its applicability, particularly for studying systemic viral infections and comprehensive host responses.

Beyond infectious disease modeling, bat organoids offer a promising platform for aging research. Bats exhibit exceptional longevity and healthy aging, featuring robust cancer resistance, efficient DNA repair, and unique immune regulation. These organoid systems could elucidate the molecular mechanisms underlying such adaptations, potentially informing healthy human aging strategies. Ultimately, continued technological innovation, including the development of a digital bat organoid platform integrated with advanced modeling and computational methodologies, may facilitate a more harmonious balance between human society and natural ecosystems, supporting proactive management of zoonotic threats and preserving biodiversity.
